# Counselling for burnout in Norwegian doctors: one year cohort study 

**DOI:** 10.1136/bmj.a2004

**Published:** 2008-11-11

**Authors:** Karin E Isaksson Rø, Tore Gude, Reidar Tyssen, Olaf G Aasland

**Affiliations:** 1 Research Institute, Modum Bad, NO-3370 Vikersund, Norway; 2 Department of Behavioural Sciences in Medicine, Institute of Basic Medical Sciences, Faculty of Medicine, University of Oslo, PO Box 1111 Blindern, NO-0317 Oslo, Norway; 3 Research Institute of the Norwegian Medical Association, PO Box 1152 Sentrum, NO-0107 Oslo, Norway; 4 Institute of Health Management and Health Economics, University of Oslo, Norway

## Abstract

**Objective** To investigate levels and predictors of change in dimensions of burnout after an intervention for stressed doctors.

**Design** Cohort study followed by self reported assessment at one year.

**Setting** Norwegian resource centre.

**Participants** 227 doctors participating in counselling intervention, 2003-5.

**Interventions** Counselling (lasting one day (individual) or one week (group based)) aimed at motivating reflection on and acknowledgement of the doctors’ situation and personal needs.

**Main outcome measures** Levels of burnout (Maslach burnout inventory) and predictors of reduction in emotional exhaustion investigated by linear regression.

**Results** 185 doctors (81%, 88 men, 97 women) completed one year follow-up. The mean level of emotional exhaustion (scale 1-5) was significantly reduced from 3.00 (SD 0.94) to 2.53 (SD 0.76) (*t*=6.76, P<0.001), similar to the level found in a representative sample of 390 Norwegian doctors. Participants had reduced their working hours by 1.6 hours/week (SD 11.4). There was a considerable reduction in the proportion of doctors on full time sick leave, from 35% (63/182) at baseline to 6% (10/182) at follow-up and a parallel increase in the proportion who had undergone psychotherapy, from 20% (36/182) to 53% (97/182). In the whole cohort, reduction in emotional exhaustion was independently associated with reduced number of work hours/week (β=0.17, P=0.03), adjusted for sex, age, and personality dimensions. Among men “satisfaction with the intervention” (β=0.25, P=0.04) independently predicted reduction in emotional exhaustion.

**Conclusions** A short term counselling intervention could contribute to reduction in emotional exhaustion in doctors. This was associated with reduced working hours for the whole cohort and, in men, was predicted by satisfaction with the intervention.

## Introduction

Research on the mental health of doctors has led to a call for preventive interventions to lower the risk of burnout and mental distress.^1^ ^2^ ^3^ ^4^ The importance of early intervention is underlined by the high prevalence of depression^5^ ^6^ and suicide^7^ ^8^ ^9^ in doctors compared with other groups and by their reluctance to seek help.^10^ ^11^ ^12^ ^13^ Doctors who work with reduced levels of functioning can be harmful to themselves, their coworkers, and patients.^1^ ^14^ ^15^ ^16^ ^17^

Early intervention programmes could ensure that practising doctors in trouble get help in time, before their problems interfere with care of patients and give rise to medical errors,^14^ ^16^ but such programmes have been poorly investigated. McCue and Sachs found reduced emotional exhaustion (one of the three dimensions in the Maslach burnout inventory) six weeks after a group intervention for young doctors,^18^ and Holt and Del Mar found reduced levels of mental distress measured with the general health questionnaire three months after they sent an intervention to general practitioners.^19^ These results emphasise the need for further long term follow-up studies to determine which factors contribute to positive changes.

Doctors in danger of reduced wellbeing or functioning might need diverse interventional approaches.^4^ ^20^ Individual (sex,^5^ ^6^ ^21^ ^22^ ^23^ age, personality traits^24^) and contextual factors (marital status, caring for children, job stress, and number of work hours^25^) contribute to risk. The impact of different types of stressors can vary with sex,^26^ and it is therefore important to investigate whether men and women benefit from different elements in an intervention.

Doctors are reluctant to seek help and often need prompting by colleagues or legislative pressure to do so.^10^ ^11^ ^12^ It is important to look at the effects of a short term intervention in doctors who are prompted to attend compared with those who attend on their own initiative.

A resource centre in Norway (Villa Sana) offers a counselling programme designed to prevent burnout, enhance mental health and quality of life, and strengthen professional awareness and identity. The self referral counselling intervention aims to motivate doctors to reflect on and acknowledge their own situation and personal needs. This might subsequently lead to them seeking medical treatment, reducing working hours, and reconsidering personal and professional priorities.^27^

In a previous cross sectional study doctors participating in this counselling programme reported a high degree of satisfaction with the intervention.^10^ ^28^ The association between satisfaction and outcome is fairly high.^29^

In a previous study we found that doctors entering the programmes had significantly higher levels of emotional exhaustion, mental distress, and job stress than Norwegian doctors in general.^27^ We studied the same cohort and carried out assessments before, immediately after, and about one year after the intervention to examine various factors associated with burnout (see box).

**Questions examined in study**

- Will levels of burnout dimensions change from baseline to follow-up, and will levels of mental distress, job stress, treatment (with a general practitioner, with a psychotherapist, or with antidepressants), sick leave, or number of work hours per week vary in the same time span?
- How will levels of burnout, job stress, and mental distress at follow-up differ from representative data for Norwegian doctors?
- Is change in emotional exhaustion (one dimension of burnout) from baseline to follow-up:
  Predicted by age, sex, personality, route of contact, or satisfaction with the intervention?
  Associated with seeking treatment, coming back for additional counselling, being on sick leave, starting use of antidepressants, or reducing number of work hours per week during the year after the intervention?

## Methods

### Study design

We included doctors consecutively attending the resource centre for a counselling intervention from August 2003 to July 2005. They completed self reported assessments in the four weeks before (baseline) and the three weeks after (intervention satisfaction) baseline intervention. Fifty three weeks (SD 6.4, range 40-70 weeks) after the intervention, they completed follow-up questionnaires, posted in eight groups (two reminders given) from autumn 2004 to spring 2006. The data were compared with data from a survey of Norwegian doctors in 2003. Baseline data indicated that the intervention programmes reached doctors in need of help as reported previously.^27^

### Sample

The mean age of included doctors was 46.9 (45.0 among practising Norwegian doctors in 2004-5), and 48% of the sample were men (64%). More details are available elsewhere.^27^

The figure[Fig fig1] shows the flow of doctors participating in the study. Initially, 187 doctors came to the single day session and 40 came to the one week course. Of the follow-up sample of 185 doctors, within the year after baseline 19 had an additional one day session and 51 had an additional course; of these 53% returned within the first eight weeks and 91% within the first six months. Seventy (38%) doctors attending the initial intervention and 12 (6%) attending a follow-up intervention brought their spouse. The interventions were basically similar for doctors with or without spouses. We examined attendance at only one session or at two sessions during the year as a predictor for reduced emotional exhaustion. Further subdivisions of the sample, according to intervention types and combinations of interventions, resulted in small subgroups that are difficult to analyse meaningfully.

**Figure fig1:**
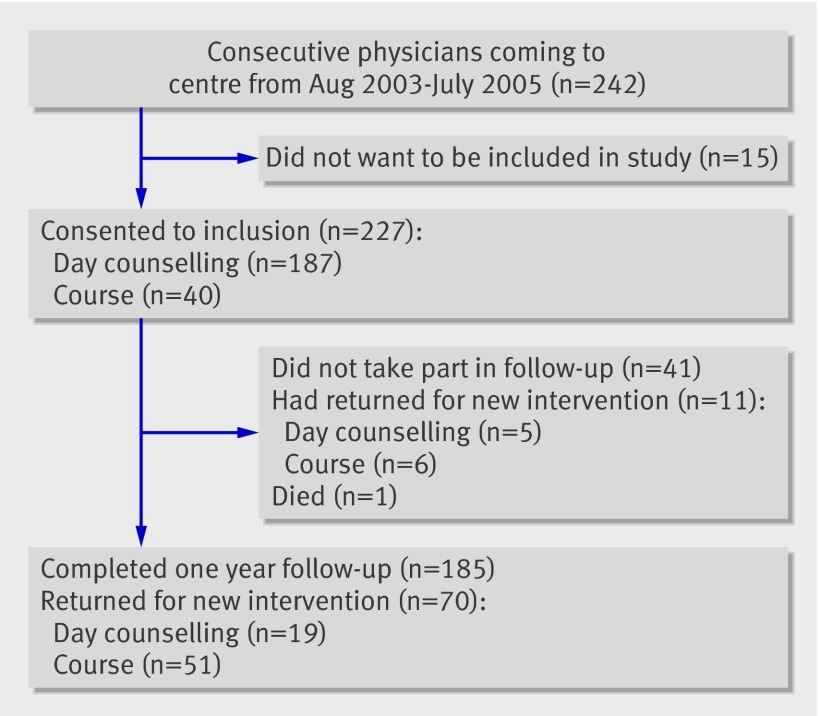
Participation in study at baseline and at follow-up

### Setting

The resource centre is open to all Norwegian doctors and was initiated in cooperation between the Norwegian Medical Association and Modum Bad psychiatric hospital. Although it is a self referral centre, some doctors reported being prompted (and some were even referred) to come by their general practitioner or another colleague.

### Intervention

The interventions were based on an integrative approach with psychodynamic, cognitive, educational, and motivational interviewing theories. Doctors chose one of two interventions.

*Single day counselling session*—Individual doctors underwent one session, lasting six to seven hours, with a psychiatrist or a specialist in occupational health. The counselling had a common structure, irrespective of counsellor. The intervention was completely confidential and did not use medical records. Doctors were invited to describe their situation including contextual factors, both related to the job and otherwise. The counsellor investigated sources of identity, self esteem, and self reliance and identified, acknowledged, and challenged present coping strategies. The doctors’ present needs in the short and longer term were focused on, and they were often advised to acknowledge these needs (for example, for treatment).

*Week long course*—These courses consisted of sessions for eight participants led by one of the same counsellors in collaboration with an occupational therapist. A daily lecture (90 minutes) introduced themes of possibilities and restraints in working life, the individuals’ resources and personality with concepts of identity, communication at work and in private, team work, and prevention of burnout. This was followed by group discussions (90 minutes) based on the participants’ own experiences, providing the opportunity to share these issues with colleagues. Doctors could do daily physical activity (75 to 360 minutes) and one session of individual counselling (60 minutes) during the week. The programme is fully described elsewhere.^27^

### Comparison sample

We compared our participating doctors with a sample of Norwegian doctors, all of whom had graduated in Norway in 1993-4. The data we used were collected at the 10 year follow-up in 2003 (n=390).^25^ ^30^ Age and sex differences were controlled for.

Our main outcome variables were changes in dimensions of burnout, mental distress, and job stress. We used regression analysis to examine prediction of change in emotional exhaustion by individual factors, satisfaction with the intervention, and changes made after the intervention, including potential confounding.

### Data at baseline and immediately after intervention

*Demographic data and personality*—We collected data on sex, age, marital status, and having children under 16 years (dichotomous variable). We used Eysenck’s personality questionnaire with six items from the neuroticism scale and six from the introvert-extrovert scale.^31^ The scales explain 82% and 83% variance, respectively, of the original scales. Cronbach’s α was 0.70 and 0.80, respectively. We scored items dichotomously (1=yes or 0=no) and obtained a sum score between 1 and 6 for each dimension, with higher scores indicating more neuroticism or being more extrovert.

*Contact with resource centre*—To determine how doctors originally contacted the centre we dichotomised answers into prompted by own general practitioner or another doctor or referred (1) and attending on own initiative (2). For the amount of contact, we dichotomised into one intervention (1) and two interventions (2)

*Satisfaction with intervention*—We used questions on satisfaction developed by the Research Institute of the Norwegian Medical Association to evaluate services at the centre after the two first years of services^28^: “How high were your expectations of the counselling session?” scored on a five point scale from very small (1) to very high (5); “Did the counselling session correspond to your expectations?” scored on a five point scale from not at all (1) to corresponded completely to expectations, including “better than expected” (5); “Did the counselling session help to clarify important factors in your situation?” scored on a four point scale from not at all (1) to a substantial degree (4); “Do you feel more able to handle your problems after the counselling session?” scored on a three point scale from no (1) to a bit more able (2), to much more able (3).

### Outcome variables at baseline and follow-up

*Burnout*—We used Maslach’s burnout inventory with three subscales: emotional exhaustion (10 items), depersonalisation/cynicism (8 items), and reduced personal accomplishment (7 items). Cronbach’s α was 0.92, 0.69, and 0.71, respectively. As in previous studies of Norwegian doctors,^32^ we used a five point scale (1=does not fit, 5=fits very well), with reference to the last two weeks at work, as the original frequency scale has been criticised for having categories that are not mutually exclusive.^33^ Personal accomplishment was presented with a reverse scale, so that high values meant low levels of accomplishment. We dichotomised emotional exhaustion into high and low, with scores above 3 indicating caseness.^32^

*Measure of mental distress*—We used Hopkins symptom checklist with five items (SCL5), which is a shortened version of SCL25 (based on SCL90R^34^), a widely used instrument designed to measure mental distress, mainly symptoms of anxiety and depression in population surveys.^34^ ^35^ The correlation between SCL5 and SCL25 was 0.91.^34^ Cronbach’s α was 0.90. The five point scale (1=not at all, 5=very much) evaluating the last two weeks has been used in previous studies of Norwegian doctors.^36^ ^37^ The measure was also dichotomised, with scores above 2.09 for men and 2.19 for women denoting cases in potential need of treatment.^27^

*Perceived job stress—*The modified version of the Cooper job stress questionnaire (Cooper, 32 items, self report,^26^ modified by Tyssen et al^38^) consisted of 17 items from a principal component analysis of data from the Norwegian student/doctor cohort^38^ in addition to nine clinically prompted items from the questionnaire.^27^ Cronbach’s α for the full 26 item instrument (job stress as reported in this paper) was 0.90, summing up scales for emotional distress (10 items), work-home interface stress (10 items), and fear of litigation (six items). Scores were given on a five point scale (1=no stress, 5=a lot of stress) with reference to the last two weeks at work.

*Sick leave*—We calculated number of weeks on full time or part time sick leave or rehabilitation or disability during the preceding year and recorded current status.

*Treatment*—We recorded if the doctor was being treated by a general practitioner, if they used antidepressants, and if they were in psychotherapy with a psychiatrist or a psychologist. 

*Working hours*—Working hours were the total hours a week of direct contact with patients, meetings, paperwork, on the telephone, research, and “other work activities.” To determine individually initiated reduction of work hours after the intervention we calculated work hours a week at baseline minus work hours a week at follow-up reported continuously and dichotomised (1=reduction, 0=no change or increase).

### Statistics

We calculated means, frequencies, and Pearson’s product moment correlations. Continuous variables were analysed with *t* test (for independent and paired samples) or Mann-Whitney U test for independent samples and Wilcoxon’s rank test for paired samples. Dichotomous variables were analysed with χ^2^ or McNemar’s test for repeated measures. We used analysis of covariance to compare means between the present sample and the representative sample of Norwegian doctors.

We tested prediction of reduction in emotional exhaustion with linear regression. In a multivariate model with sex and age we included significant bivariate associations with preceding predictors (personality dimensions, marital status, having children aged under 16, way of contact, satisfaction with the intervention) and concurrent predictors (such as starting psychotherapy, taking sick leave, starting antidepressants, reduced working hours, and returning for an additional counselling session during follow-up). We examined interactions between sex and significant predictors and also performed separate analyses by sex. Results are reported as standardised β values.

### Missing data

The variables for satisfaction with the intervention were missing for 34 individuals. There were no significant differences in demographic or distress variables between the groups with and without missing variables so inclusion should not reduce the internal validity.

When one or a few items were missing in an instrument we used the mean score of completed items. Some of the items in the Maslach burnout inventory and in job stress were not relevant for all respondents because of differences in working conditions (not working directly with patients such as in laboratory work, leadership, research). Mean scores of remaining items were used. Instruments with all items missing reduced the number in the relevant analyses (this concerned three to four instruments at baseline and four to 15 instruments at follow-up). For all analyses we used SPSS 15.0.

## Results

The cohort comprised 227 doctors (94% of 242 eligible). Of these, 88 men and 97 women (185, 81%) completed follow-up, three did not want to be assessed, one had died, and 38 gave no response (figure).[Fig fig1]

Table 1 shows the demographic variables, personality dimensions, route of attendance, and satisfaction with the intervention for doctors who completed follow-up[Table tbl1]. The expectations of the intervention were generally high: 52/149 (35%) had medium high, 80/149 (54%) quite high, and 17/149 (11%) very high expectations. Further baseline data, including a comparison with Norwegian doctors in general, have been presented previously.^27^

**Table 1 tbl1:** Individual variables, route of attendance, personality dimensions, and satisfaction with intervention for 185 doctors after participation in counselling intervention for burnout. Figures are means (SD) or numbers of participants (%, 95% confidence interval)

	Men (n=88)	Women (n=97)
Age (years)	49.4 (9.0)	44.7 (8.2)
Married/cohabiting	76/88 (86.4, 79.2 to 93.6)	74/97 (76.3, 66.8 to 86.0)
Have children aged <16 years	38/88 (43.2, 32.9 to 53.5)	47/97 (48.5, 38.6 to 58.4)
Prompted by doctor to come to counselling	27/88 (30.7, 21.1 to 40.3)	42/97 (43.3, 33.4 to 53.2)
Personality dimension:		
Neuroticism (1-6)	2.85 (1.87)	2.51 (1.76)
Extroversion (1-6)	3.41 (2.16)	3.98 (1.87)
How high were expectations to counselling session? (1-5)	3.77 (0.66)*	3.72 (0.70)†
Were expectations to counselling session fulfilled? (1-5)	4.50 (0.61)*	4.44 (0.63)†
Counselling contributed to clarify important factors (1-4)	3.63 (0.64)*	3.53 (0.57)†
Felt more able to handle problems after counselling (1-3)	2.39 (0.49)*	2.37 (0.56)†

*n=69/70.

†n=79/81.

We found no significant differences in age, sex, or stress levels at baseline between those who completed and those who did not complete follow-up assessments (table 2). A higher proportion of those who did not complete follow-up were taking antidepressants at baseline.

**Table 2 tbl2:** Comparison between doctors at baseline who completed and who did not complete follow-up. Figures are means (SD) or numbers of participants (%; 95% confidence interval)

	Completed follow-up (n=181-185)	Did not complete follow-up (n=41-42)	Level of significance
Age (years)	47.0 (8.9)	46.8 (9.5)	−0.07,* P=0.94
Men	88/185 (47.6; 40.0 to 54.8)	22/42 (52.4; 37.3 to 67.5)	0.32,† P=0.57
SCL5 (1-5)	2.83 (1.09)	3.06 (1.15)	1.18,* P=0.24
Maslach burnout inventory:			
Emotional exhaustion (1-5)	3.03 (0.94)	3.25 (0.94)	1.37,* P=0.17
Depersonalisation (1-5)	1.90 (0.56)	2.00 (0.56)	1.06,* P=0.29
Reduced personal accomplishment (1-5)	2.30 (0.51)	2.19 (0.50)	−1.24,* P=0.21
Total job stress (1-5)	2.42 (0.71)	2.52 (0.77)	0.85,* P=0.39
Use of antidepressants	35/183 (19; 13.3 to 24.7)	16/41 (38; 23.1 to 52.9)	7.01,† P=0.008

*t test for continuous variables

†χ^2^ for dichotomous variables.

Levels of burnout (emotional exhaustion), mental distress (SCL5), and job stress were significantly lower at one year follow-up than at baseline (table 3). There were no significant differences in these changes according to sex, route of attendance, or with attending one or two interventions during the year. There was a significant reduction in the proportion of the cohort above levels indicating burnout in relation to emotional exhaustion and need for treatment in relation to SCL5 at follow-up (table 3).[Table tbl3]

**Table 3 tbl3:** Burnout, mental distress, and job stress at baseline and at one year follow-up in cohort of doctors participating in counselling for burnout. Figures are means (SD) or numbers of participants (%; 95% confidence interval)

	Baseline	One year follow-up	Difference	Paired *t* test	P value
Maslach burnout inventory:					
Emotional exhaustion (1-5) (n=168)	3.00 (0.94)	2.53 (0.76)	0.47 (0.91)	6.76	<0.001
Emotional exhaustion: score indicates caseness (>3)	76 (45.2; 37.7 to 52.7)	41 (24.4; 17.9 to 30.0)	35 (20.8; 14.7 to 26.9)	—	<0.001*
Depersonalisation/cynicism (1-5) (n=166)	1.90 (0.57)	1.75 (0.52)	0.16 (0.50)	4.02	<0.001
Reduced personal accomplishment (1-5) (n=168)	2.29 (0.49)	2.25 (0.52)	0.03 (0.51)	0.80	0.43
SCL5 (Hopkins symptom checklist:					
Score (1-5) (n=181)	2.84 (1.08)	2.07 (0.92)	0.77 (0.96)	10.73	<0.001
Score indicates caseness	131/181 (72.3; 65.8 to 78.8)	73/181 (40.3; 33.1 to 47.4)	58/181 (32; 25.2 to 38.8)	—	<0.001*
Total job stress:					
Score (1-5) (n=177)	2.40 (0.70)	1.97 (0.61)	0.43 (0.67)	8.52	<0.001

*McNemar.

The proportion of doctors who had undergone psychotherapy increased substantially, from 20% at baseline to 53% in the year after the intervention (table 4). The number of weeks on sick leave in the preceding year and proportion of physicians receiving disability or rehabilitation benefits increased after the intervention, whereas the proportion of physicians on current full time sick leave was substantially lower at follow-up (6% *v* 35%, table 4).[Table tbl4] A higher proportion of women were on full time sick leave at baseline: 35/86 (41%, 95% confidence interval 31% to 51%) *v* 18/80 men (22%, 10% to 26%), P=0.01. There were no significant sex differences at follow-up.

**Table 4 tbl4:** Proportion of doctors receiving treatment and on sick leave at baseline and follow-up, number of weeks of sick leave during preceding year, and working hours among those participating in counselling for burnout. Percentages with 95% confidence intervals shown in parentheses

	Baseline	One year follow-up	P value
No (%) (McNemar’s test):			
In treatment with general practitioner at present	68/173 (39.3; 32.0 to 46.6)	69/173 (39.9; 32.6 to 47.2)	0.991
In psychotherapy at some point during past year	36/182 (19.8; 14.0 to 25.6)	97/182 (53.3; 46.1 to 60.1)	<0.001
Taking antidepressants	34/175 (19.4; 13.5 to 25.3)	31/175 (17.7; 12.0 to 23.4)	0.72
Full time sick leave	63/182 (34.6; 27.4 to 41.2)	10/182 (5.5; 2.2 to 8.8)	<0.001
Part time sick leave	7/182 (3.8; 1.0 to 6.6)	11/182 (6.0; 2.5 to 9.5)	0.48
Full time disability/rehabilitation benefits	1/182 (0.5; −0.5 to 1.5)	10/182 (5.5; 2.2 to 8.8)	0.004
Part time disability/rehabilitation benefits	3/182 (1.6; −0.2 to 3.4)	2/182 (1.1; −0.4 to 2.6)	0.99
Mean (SD) (Wilcoxon rank test):			
No of weeks on full time sick leave in preceding year (n=162)*	4.0 (6.9)	8.0 (13.7)	z=−3.39, 0.001
No of weeks on full time sick leave/disability or rehabilitation benefits in preceding year (n=167)	4.4 (7.9)	8.5 (14.4)	z=−3.29, 0.001
Working hours/week (n=165)	43.6 (7.9)	42.0 (12.1)	z=2.25, 0.03
Working hours/week for doctors who reduced working hours after baseline (n=81)	46.0 (7.6)	37.7 (10.9)	z=−7.82, <0.001
Working hours/week for doctors who did not reduce working hours after baseline (n=84)	41.3 (7.6)	46.0 (11.9)	z=6.75, <0.001

*Not including those on disability/rehabilitation benefits or retired at follow-up.

The numbers of hours worked a week in the cohort at baseline was not significantly different from that of the representative survey of doctors in 2003^25^ (43.4 (SD 7.90) *v* 43.6 (SD 8.14), P=0.12). Participating doctors reduced their working hours by a mean of 1.6 hours a week at follow-up (table 4).[Table tbl4] Compared with women, men worked a higher average number of hours a week at baseline: 45.1 hours (SD 7.58) *v* 42.0 hours (SD 7.94) (P=0.01). This difference disappeared at follow-up.

Compared with Norwegian doctors surveyed in 2003, the follow-up sample had lower or non-significantly different levels of emotional exhaustion and total job stress but still had significantly higher values on the Hopkins symptom checklist, adjusted for age and sex (table 5).

**Table 5 tbl5:** Doctors at one year follow-up compared with Norwegian doctors surveyed in 2003, controlled for sex and age. Figures are means of estimated marginal (95% confidence interval of standard error)

	Doctors at follow-up (n=170/185)	Norwegian doctors 2003 (n=390)	*F*, P value
Emotional exhaustion (1-5)	2.52 (2.39 to 2.65)	2.47 (2.38 to 2.57)	F=0.24, 0.62 (0.05*)
SCL5 (1-5)	2.12 (1.99 to 2.49)	1.54 (1.43 to 1.64)	F=39.56, <0.001
Total job stress (1-5)	1.99 (1.89 to 2.09)	2.20 (2.13 to 2.28)	F=9.54, 0.002 (0.01*)

*For independent sex effect.

We found significant bivariate associations for the whole sample between reduction in emotional exhaustion and a linear function of age (β=−0.16, P=0.04; younger doctors have a greater reduction), neuroticism (β=0.29, P<0.001), extroversion (β=−0.23, P=0.003; more introversion gives more reduction), and reduction of work hours (β=0.21, P=0.01). The significant effects maintained in the multivariate model were from neuroticism, with 8.7% explained variance (β=0.23, P=0.005), extroversion, with an additional 3.7% explained variance (β=−0.20, P=0.01), and reduction of work hours, with an additional 2.7% explained variance (β=0.17, P=0.03), n=158.

There were no interactions between sex and significant predictors. Among men, reduction in emotional exhaustion was associated with neuroticism (β=0.39, P<0.001), extroversion (β=−0.32, P=0.004), satisfaction with the intervention (“did the counselling session correspond to your expectations?”) (β=0.27, P=0.04), and reduction of work hours (β=0.25, P=0.03). The significant effects maintained in the multivariate model were from neuroticism, with 17.5% explained variance (β=0.32, P=0.02), and satisfaction with the intervention, with an additional 6.5% explained variance (β=0.25, P=0.04), n=58.

There were no bivariate associations among women.

## Discussion

### Principal findings

One year after a counselling intervention initially stressed doctors reported a reduction in emotional exhaustion (burnout) and job stress to the levels found in a representative sample of Norwegian doctors. The substantial reduction in the proportion of those in need of treatment according to SCL5 and in burnout (emotional exhaustion) underlines the clinical relevance of this improvement in wellbeing. After adjustment for sex, age, and the personality dimensions of neuroticism and introversion, reduced emotional exhaustion was associated with “reduction of work hours” after the intervention in the whole cohort. Among men reduction in emotional exhaustion was also predicted by satisfaction with the intervention (that is, “the counselling intervention corresponded to my expectations,” where reported expectations generally were high), indicating that the observed reduction in distress could be related to the intervention.

The considerable reduction in full time sick leave at follow-up compared with baseline, together with a relatively modest reduction in weekly work hours (less than two hours), supports the notion that the intervention had a positive impact on the working capacity of the doctors. Relatively small adjustments might thus have had considerable impact on their mental health and functioning at work. Further investigation could determine whether this was caused by, or mediated through, the increase in number of weeks on sick leave after the intervention or the fact that more doctors sought psychotherapy.

### Strengths and weaknesses

The longitudinal design with one year follow-up and the comparison with a representative sample of Norwegian doctors are strengths in this study. The high proportion of participants who completed follow-up (81%) strengthens the internal reliability of the results.

The validity of the self reported findings is strengthened by our adjustment for neuroticism, which can influence the perception of distress variables towards reporting negatively (so called negative affectivity).^39^

The non-significant differences in reduction of distress parameters for doctors prompted (“referred”) by a colleague compared with attending on their own initiative strengthens the generalisability of the results as previous studies show that doctors often need prompting or referral to health services.^3^ ^40^ ^41^

The study has several limitations, principally the opportunistic design that impedes determination of a causal relation to the intervention, because of spontaneous regression towards the mean, or to other factors not assessed in this study. The regression analysis, however, indicates an association between intervention and outcome.

We did not find any association between the change in stress levels and attending an additional intervention during follow-up, which increases the generalisability of the study. The lack of further analyses of subgroups according to intervention types and combinations of interventions could, however, give a false negative finding (type II error). The regression analyses for each sex are also subject to possible type II errors. Near significant associations found should be studied in larger samples to ascertain sex differences. Recall bias, especially concerning sick leave and working hours during the preceding year, could be an issue, and objective measures could have given more accurate estimates. More of the participants lost to follow-up reported taking antidepressants at baseline, but as this does not correspond with significantly higher distress rates at baseline we consider it of minor importance to the results.

### Strengths and weaknesses in relation to other studies

Previous intervention studies have shown a reduction in emotional exhaustion and in scores on the general health questionnaire after short follow-up periods (six weeks, three months), and they describe educational group interventions,^18^ ^19^ whereas the programmes we studied used individual counselling (as well as group sessions) and a one year follow-up period. Comparisons are therefore difficult.

High expectations and demands of capacity and ability to cope, combined with a low acceptance of personal needs,^5^ ^42^ has led to a reluctance among doctors to seek treatment, especially for mental distress.^10^ ^11^ ^23^ ^26^ In accordance with this, a study of a self referral specialist service for doctors in England^43^ as well as the cross sectional study of our programmes^27^ suggest that such services can lead to earlier engagement, thus “enhancing the possibility of nipping problems in the bud.”^43^ The increase after the intervention in treatment seeking indicates increased acknowledgment (possibly perception) and acceptance of personal needs, and perception of high levels of emotional distress has been shown to predict professional help seeking.^44^

The personality dimensions of neuroticism and introversion predict burnout in doctors^24^ ^45^ but are also important for functioning well. Self criticism, closely related to neuroticism, is associated both with depressive symptoms as well as with a higher capacity for empathy.^46^ Support related to these personality dimensions would thus help doctors towards sound mental health in combination with good performance and empathic ability.^47^

The relation between work hours and perceived stress is ambiguous in Norwegian studies.^10^ ^25^ ^38^ Work hours vary between countries, with longer average hours in the United States^48^ and in the United Kingdom than in Norway. A potential mediator between work hours and burnout has been suggested to be the “fit,” defined as “the extent to which workers realize . . . their plans for optimizing their own work—and non-work needs.”^49^ Fit will necessarily be influenced by the norms of the particular society. This could explain the positive impact of reduced working hours on reduction in emotional exhaustion as seen in the US^48^ ^50^ after the implementation of duty hour standards as well as after the individually initiated reduction in work hours found in our study.

Male doctors have previously reported more stress caused by job demands,^26^ whereas female doctors have reported more stress related to the work-home interface,^26^ ^51^ and women value social support at work more.^52^ The sex differences regarding the importance of reduced working hours and satisfaction with the intervention (among men but not among women) could indicate that the chosen outcome variables did not adequately reflect the more complex matters involved in assessing the relation between the observed reduction in emotional exhaustion and multiple factors in the work-home interface^51^ and social relations at work.^25^ Further investigations of sex differences are important to tailor relevant interventions.

### Possible explanations and implications

Our findings indicate that seeking a counselling intervention could be conducive to reduction of burnout among doctors. Considering doctors’ reluctance to seek help, despite high levels of distress, it is important to offer interventions that facilitate access and that can enhance motivation to reconsider personal and professional priorities when necessary.

### Unanswered questions and future research

This study is a preliminary study examining the associations between the alleviation of burnout and a counselling intervention. The indications of factors possibly contributing to reduction in emotional exhaustion need to be further investigated with a more controlled design.

**What is already known on this topic**

Previous research on doctors’ mental health has documented need for interventions to prevent burnout and distress
Few evaluations of such interventions have been published, and follow-up times have been short

**What this study adds**

A short term counselling intervention could contribute to reduction of emotional exhaustion (one dimension of burnout)
Reduction in emotional exhaustion was associated with reduction in working hours, and, among men, satisfaction with the intervention predicted reduction in exhaustion

## Notes

We thank the participating doctors for their time and engagement in registering data. We also thank Per Vaglum, professor emeritus at the Department of Behavioural Sciences in Medicine, University of Oslo, for substantial and valuable comments during manuscript revision and John Boettiger, professor emeritus, for language revision.

Ethical approval: The study was approved by the data inspectorate through the Norwegian Social Science Data Services. The regional ethical research committee in the south of Norway did not consider consent necessary for this study. All doctors gave written informed consent.

Provenance and peer review: Not commissioned; externally peer reviewed.

## Notes

**Cite this as: ***BMJ* 2008;337:a2004
